# Presumed Symbolic Use of Diurnal Raptors by Neanderthals

**DOI:** 10.1371/journal.pone.0032856

**Published:** 2012-03-05

**Authors:** Eugène Morin, Véronique Laroulandie

**Affiliations:** 1 Department of Anthropology, Trent University, Peterborough, Ontario, Canada; 2 CNRS, PACEA, UMR 5199, Université Bordeaux, Talence, France; Illinois State University, United States of America

## Abstract

In Africa and western Eurasia, occurrences of burials and utilized ocher fragments during the late Middle and early Late Pleistocene are often considered evidence for the emergence of symbolically-mediated behavior. Perhaps less controversial for the study of human cognitive evolution are finds of marine shell beads and complex designs on organic and mineral artifacts in early modern human (EMH) assemblages conservatively dated to ≈100–60 kilo-years (ka) ago. Here we show that, in France, Neanderthals used skeletal parts of large diurnal raptors presumably for symbolic purposes at Combe-Grenal in a layer dated to marine isotope stage (MIS) 5b (≈90 ka) and at Les Fieux in stratigraphic units dated to the early/middle phase of MIS 3 (60–40 ka). The presence of similar objects in other Middle Paleolithic contexts in France and Italy suggest that raptors were used as means of symbolic expression by Neanderthals in these regions.

## Introduction

In recent years, several studies have argued that early (≈100–70 ka) occurrences of marine shell beads (mostly *Nassarius* specimens) and fragments of ocher pigments in Israel, the Maghreb and South Africa indicate that late Middle and early Late Pleistocene EMHs were capable of symbolically-mediated behavior [1]–[4]. In South Africa, engraved motifs on ocher and bone at Blombos and on ostrich eggshell fragments at Diepkloof in contexts dated between 77 and 60 ka supports this view [4], [5]. In contrast, debates are more vivid concerning whether comparatively complex activities were common in Neanderthals [6]–[8]. In Europe, ocher was widely utilized, allegedly as colorant, during the Middle Paleolithic [9], whereas evidence for the ornamental use of pigment-stained marine shells is possibly present at Cueva de los Aviones (≈50 ka) and Cueva Antón (≈40 ka) in Spain [6]. However, few studies have investigated the non-alimentary use of birds during the late Middle and early Late Pleistocene. Here we present new archaeological evidence relevant to the debate on the emergence of symbolic thought.

In Europe and southwest Asia, marks of human activity are rare on bird bones before the Upper Paleolithic, which suggests that this class of prey species was seldom eaten or utilized [10]–[12]. However, there are two notable exceptions to this pattern. The sequence (MIS 9–5e) of Cova Bolomor in eastern Spain provides a relatively unique example for the Middle and early Late Pleistocene of human consumption of small- to large-sized ground-feeding birds (passerines, corvids, pigeons, Galliformes) and waterfowl (Anatidae), attested by cutmarks or human tooth marks on meat-bearing elements and anthropogenic bone fractures [13]. The patterns of bird consumption at Cova Bolomor are noteworthy because they are reminiscent of those documented considerably later during the Upper Paleolithic [10], [11]. The late Mousterian (45–40 ka) avifaunal samples from Grotta di Fumane in Italy differ from Cova Bolomor in showing cutmarks on bones of medium- (red-footed falcon *Falco vespertinus*) and large-sized raptors (golden eagle *Aquila chrysaetos*, lammergeier *Gypaetus barbatus*, Eurasian black vulture *Aegypius monachus*). Although cutmarks were also observed on non-raptorial species (Alpine chough *Pyrrhocorax graculus*, common wood pigeon *Columba palumbus*), the over-representation of raptors in the cutmark sample and the anatomical distribution of these marks—all are found on wing and foot bones—suggest a symbolic, rather than alimentary, use of bird parts by Neanderthals [7]. The data that we present here provide additional support for symbolically-mediated behavior in this population.

## Methods

All the unidentified and taxonomically identified bird specimens from Combe Grenal and Les Fieux were inspected for cutmarks using a stereomicroscope at low magnification. The cutmarks that we identified are unambiguous and fall well within the range of those documented in ungulate taxa [12]. The cutmarks are often deep and tend to show sharp boundaries typical of incision marks produced with a stone tool. The anatomical terms in this paper follow the nomenclature currently used in the analysis of bird remains.

## Results

**Figure 1:**
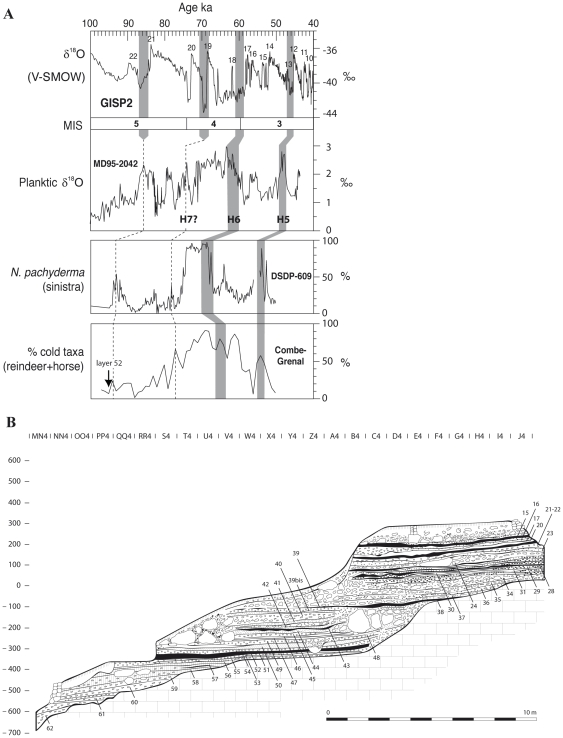
The archaeological context at Combe-Grenal. A) tentative correlations between the percentage of cold-adapted ungulate taxa (reindeer *Rangifer tarandus* and horse *Equus ferus caballus*) in layers 55–1 at Combe-Grenal and variations in two marine proxies: the percentage of planktic foraminifer *Neogloboquadrina pachyderma* in the DSDP-609 core [16] and fluctuations in planktic δ^18^O in the MD95–2042 core [17]. The series are compared with Dansgaard-Oschger events 10–22 in the GISP2 ice core following ref. [18]. H5, H6, and H7 are Heinrich events. B) the stratigraphy at Combe-Grenal (modified from ref. 14). Layer 52 is the lowermost black layer. The percentages of reindeer and horse in A) were calculated using ungulate counts from refs. [19] and [20] (elephantids, indeterminate cervids, and possible *Megaloceros* remains excluded). The percentages of reindeer and horse specimens are for individual layers, except for layers 55–54, 49–48, 46–44, 43–42, 16–15, 5–4 and 2–1, which constitute aggregates of two or three occupations in order to increase sample size.

Combe Grenal is a Middle Paleolithic site in the Dordogne region of France with a remarkably long (13 m) sequence comprising sixty-five stratigraphic layers [14]. Of relevance to this paper are the more recent human occupations (55–1) from this sequence, which span from MIS 5b to the first half of MIS 3 [refs. 12], [15]. Correlations between the taxonomic representation of cold-adapted ungulate species at Combe-Grenal and marine proxies in the DSDP-609 and MD95–2042 cores (North Atlantic) suggest that layer 52—an occupation with evidence of human use of birds—was deposited ≈90 ka (Figure 1a). The lithic assemblage from this layer is attributed to the Typical Mousterian [14], whereas the human-accumulated faunal assemblage is dominated, in ascending order, by horse (*Equus ferus caballus*), roe deer (*Capreolus capreolus*) and red deer (*Cervus elaphus*) [15], [19]. The position of layer 52 near the bottom of the sequence (Figure 1b) and the lack of Upper Paleolithic occupations at Combe-Grenal largely exclude the possibility of mixing with post-Mousterian materials.

**Figure 2:**
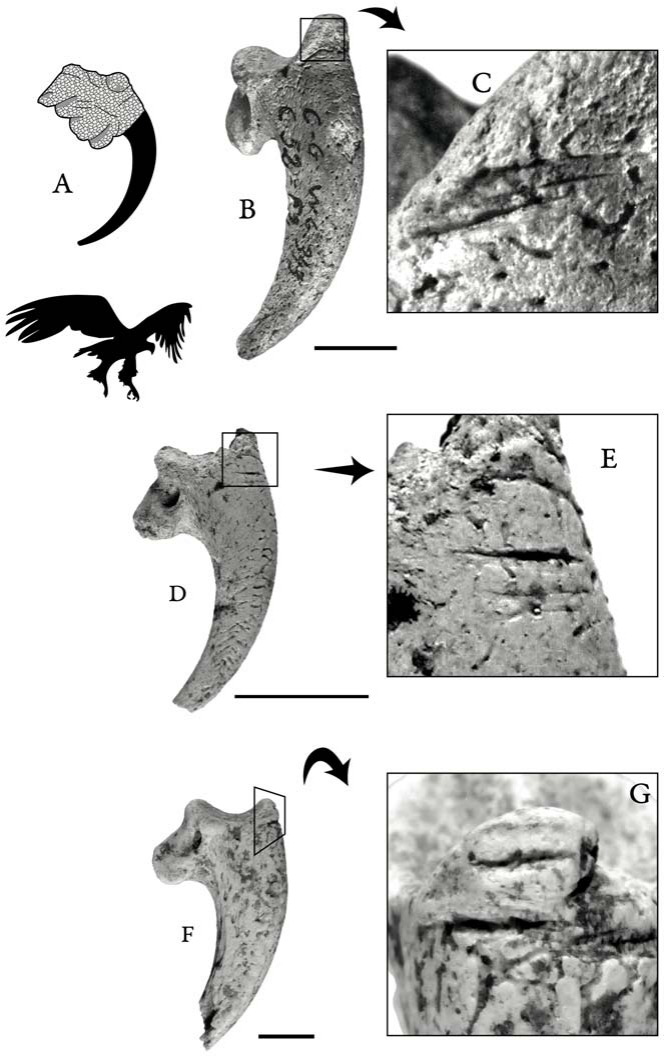
Stone tool incisions on terminal phalanges of diurnal raptors from Middle Paleolithic occupations in France. A) example of a fully fleshed golden eagle digit. B–G show cutmarked terminal phalanges from layer 52 at Combe-Grenal (B–C, golden eagle) and layers Jbase (D–E, white-tailed eagle) and I/J (F–G, white-tailed eagle) at Les Fieux. The black bars correspond to 1 cm. Philippe Jugie took the Combe-Grenal photographs, the others were taken by V.L.

Samples of identified bird remains are relatively small at Combe-Grenal [21]. This may partly reflect collection bias, given that faunal remains were selectively recovered at the site. Layer 52 contains a modest sample (*n* = 7) of bird bones, all but one assigned to small indeterminate species. The only taxonomically identified bird remain in this sample is a terminal phalanx of a golden eagle (*Aquila chrysaetos*). This well-preserved specimen bears on its proximo-dorsal side two incisions produced by a stone tool. These incisions closely coincide with the proximal margin of the keratinous sheath overlying the terminal phalanx of the digit, which suggests removal of the claw sheath (Figure 2a–c). The absence of other parts of raptors in this layer and the fact that bird claws are predominantly made of a tough fibrous protein called β-keratin [22] point to a non-alimentary use of an eagle claw.

The presence of tool marks on a raptor terminal phalanx is not unique to Combe-Grenal. We have identified similar marks at Les Fieux, a cave site in southwestern France. These specimens consist of two terminal phalanges of a white-tailed eagle (*Haliaetus albicilla*), one from stratigraphic unit Jbase and the other from the possibly coeval unit I/J (Figure 2d–g). Although chronometric dates are lacking for these units, a study of micro-mammalian remains indicates that the Les Fieux eagle phalanges are of MIS 3 age [23]. This is supported by the composition of the microfaunal sample and the lack of archaic species (Jeannet, pers. comm. 2012). Consequently, and because they are associated with Middle Paleolithic industries—more specifically, the Mousterian of Acheulean Tradition for units Ks and Jbase [24] and the Denticulate Mousterian for unit I/J [25]—the phalanges likely date to between 60 and 40 ka. Despite the limited data, this chronological proposition is in agreement with a recent synthesis of the Mousterian industries of France [26]. The eagle phalanges from units Jbase and I/J exhibit cutmarks in a similar anatomical location as those on the considerably older (by 30 thousand years or more) specimen from Combe-Grenal. Similar cutmarks on raptor terminal phalanges are also documented at two other Mousterian sites: Pech de l'Azé IV, France (in a layer dated to ≈100 ka) [27], and Grotta di Fumane, Italy (in a layer dated to ≈44 ka) [7]. Because the raptor specimens described here belong to four sites and are from occupations dated between 100 and 44 ka, it seems reasonable to argue that Neanderthals in France and Italy regularly used terminal phalanges of birds of prey during the Middle Paleolithic.

The selective use of raptors by at least some Neandertal groups is confirmed by taxonomic data. According to Table 1, the sample of Mousterian bird specimens with cutmarks in France and Italy is strongly dominated by medium- and large-sized diurnal raptors (15/21 or 71.4%). The percentage of diurnal raptors in this sample is significantly greater than in the rare pre-Upper Paleolithic assemblages attributed to human foraging activities, such as those of Cova Bolomor in Spain, where birds of prey are absent from the cutmark sample (0/60 or 0%, χ^2^ = 52.6, *p*<.0001, all levels combined). Moreover, tool marks on ground-feeding (Phasianidae) and water-adapted (e.g., ducks, geese, swans) birds are poorly represented in the French-Italian cutmark sample (1/21 or 4.8%). These taxa, which became economically important during the Upper Paleolithic [10]–[12], are significantly more abundant in the cutmark sample from Cova Bolomor (43/60 or 71.7%, χ^2^ = 28.1, *p*<.0001). Together, these patterns attest to a non-subsistence oriented use of diurnal raptors by Neanderthals during the Middle Paleolithic of France and Italy.

**Table 1:** List of late Middle and Late Pleistocene sites from Europe that comprise bird remains with unambiguous anthropic marks.

Site, period		Taxon and part with cutmarks	Date (ka)	Cutmarks(*n*/NISP taxon, %)		Reference
	C. Bolomor, XVIIc	Pass., Phasianidae, *Anas* sp.	350–300	6/35	17.1	[13]
	” , XII	mute swan *Cygnus olor*, Gal., *Anas* sp.	180	4/30	13.3	”
	” , XI	*Aythya* sp. (diving ducks)	<150	18/202	8.9	”
	” , IV	Pass., corvids, Gal., *Columba* sp., Anat.	>120	32/209	15.3	”
Germany	Salzgitter-Lebenstedt	*Cygnus* sp. (swan), cmtc	MIS 3?	1/?	-	[28]
		*Anas* sp. (dabbling ducks), humerus	”	1/?	-	”
France	Lazaret CII	rock dove *Columba livia*, humerus	190–150	1?/12288	0	[29]
	Pech de l'Azé IV, 8	medium-sized raptor, pha	100	1/?	-	[27]
	Combe Grenal, 52	golden eagle *A. chrysaetos*, pha	90	1/1	100	this study
	Gr. de l'Hyène, Arcy	”	MIS 3?	1/?	-	[7]
	Pech de l'Azé I, 4	golden eagle *A. chrysaetos*, pha (n = 2)	MIS 3	2/3	67.0	[30]
	Baume de Gigny, XV	whooper swan *Cygnus cygnus*, pha	50?	1/1	100	[21]
	Les Fieux, Kdentic.	golden eagle *A. chrysaetos*, femur	MIS 3	1/1	100	[25]
	” , Ks	white-tailed eagle *H. albicilla*, pha	”	1/3	33.3	this study
	” , ”	black vulture *Ae. monacus*, pha	”	1/1	100	”
	” , ”	common raven *Corvus corax*, tibia	”	1/21	4.8	”
	” , Jbase	white-tailed eagle *H. albicilla*, pha	”	1/1	100	”
	” , I/J	”	”	1/3	33.3	”
	Le Noisetier	*Falco* sp. (falcon), humerus	”	1/1	100	”
Italy	Fumane, A12	golden eagle *A. chrysaetos*, pha	”	1/?	-	[7]
	” , A9	black vulture *Ae. monachus*, cmtc	”	1/?	-	”
	” , A6–A5	lammergeier *Gypaetus barbatus*, ulna	40–45	1/15	6.7	”
	” , ”	red-footed falcon *Falco v.*, humerus	”	1/1	100	”
	” , ”	wood pigeon *C. palumbus*, cmtc	”	1/103	1.0	”
	” , ”	alpine chough *P. graculus*, ulna	”	2/27	7.4	”

Abbreviations: Pass = Passerines; Gal = Galliformes; Anat. = Anatids; *A.* = *Aquila*; *H.* = *Haliaetus*; *Ae.* = *Aegypius*; *C*. = *Columba*; *P.* = *Pyrrhocorax*; *v.* = *vespertinus*; pha = pedal phalanx; cmtc = carpometacarpus.

Post-Middle Paleolithic sites are excluded. A dash in a cell indicates a lack of data. Parts are not listed at Cova Bolomor for the sake of brevity.

## Discussion

Because claws are inedible, the specimens presented here are not compatible with human consumption. This means that the tool-marked terminal phalanges found at Combe-Grenal, Les Fieux, Pech de l'Azé IV, and Grotta di Fumane were likely used as tools and/or as items of symbolic expression. Although the sample size is small, the fact that all the terminal phalanges that show cutmarks are from eagles argues against their utilization in strictly non-symbolic contexts. This last pattern is noteworthy because eagles are among the rarest birds in the environment, a pattern explained by their high trophic position in the food web [31]. This bias toward large and powerful diurnal raptors possibly indicates that the claws were used in symbolically-oriented contexts by Neanderthals, although the latter contexts remain to be more precisely defined. One possibility is that they were used as ornaments, as has been suggested for the Upper Paleolithic occupations (dated to *ca*. 20 ka) at Meged Rockshelter in Israel [32].

These results do not exclude occasional consumption by archaic humans of large diurnal raptors. Two previously unpublished Mousterian specimens (one proximal femur of white-tailed eagle from Les Fieux and one proximal humerus from an indeterminate falcon at Grotte du Noisetier) present cutmarks on meat-bearing portions (Table 1). Although the tool marks—particularly the one on the femur—are congruent with meat consumption, the small sample size limits the significance of these observations with respect to diet.

These results cast additional light on Neandertal behavioral adaptation by suggesting the rise in this population of complex cognitive abilities similar to those of coeval EMH. Moreover, the use of raptor terminal phalanges during several temporal phases of the French Mousterian may indicate continuity in behavior in this region, although the possibility of simple convergence cannot be excluded. More research on bird remains will be required to fully assess the implications of these patterns.

## References

[1] Marean CW, Bar-Mathews M, Bernatchez J, Fisher E, Goldberg P (2007). Early human use of marine resources and pigment in South Africa during the Middle Pleistocene.. Nature.

[2] Henshilwood CS, d'Errico F, van Niekerk KL, Coquinot Y, Jacobs Z (2011). A 100,000-year-old ochre-processing workshop at Blombos Cave, South Africa.. Science.

[3] Vanhaeren M, d'Errico F, Stringer C, James SL, Todd JA (2006). Middle Paleolithic shell beads in Israel and Algeria.. Science.

[4] Henshilwood CS, Dubreuil L (2011). The Still Bay and Howiesons Poort, 77–59 ka: Symbolic material culture and the evolution of the mind during the African Middle Stone Age.. Curr Anthropol.

[5] Texier PJ, Porraz G, Parkington J, Rigaud JP, Poggenpoel C (2010). A Howiesons Poort tradition of engraving ostrich eggshell containers dated to 60,000 years ago at Diepkloof Rock Shelter, South Africa.. Proc Natl Acad Sci USA.

[6] Zilhão J, Angelucci DE, Badal-García E, d'Errico F, Daniel F (2010). Symbolic use of marine shells and mineral pigments by Iberian Neanderthals.. PNAS.

[7] Peresani M, Fiore I, Gala M, Romandini M, Tagliacozzo A (2011). Late Neanderthals and the intentional removal of feathers as evidence from bird bone taphonomy at Fumane Cave 44 ky B.P., Italy.. PNAS.

[8] Mellars P (2010). Neanderthal symbolism and ornament manufacture: The bursting of a bubble?. PNAS.

[9] Soressi M, d'Errico F, Vandermeersch B, Maureille B (2007). Pigments, gravures, parures: Les comportements symboliques controversés des Néandertaliens.. Les Néandertaliens. Biologie et cultures.

[10] Stiner MC, Munro ND, Surovell TA (2000). The tortoise and the hare: Small-game use, the broad-spectrum revolution and paleolithic demography.. Curr Anthropol.

[11] Laroulandie V, Brugal JP, Desse J (2004). Exploitation des ressources aviaires durant le Paléolithique en France: Bilan critique et perspectives.. Petits animaux et sociétés humaines. Du complément alimentaire aux ressources utilitaires.

[12] Morin E (2012). Reassessing Paleolithic subsistence: The Neandertal and modern human foragers of Saint-Césaire, France.

[13] Blasco R, Fernández Peris J (2012). A uniquely broad spectrum diet during the Middle Pleistocene at Bolomor Cave (Valencia, Spain).. Quat Int.

[14] Bordes F (1972). A tale of two caves.

[15] Delpech F (1996). L'environnement animal des Moustériens Quina du Périgord.. Paléo.

[16] Allen JRM, Brandt U, Brauer A, Hubberten HW, Huntley B (1999). Rapid environmental changes in southern Europe during the last glacial period.. Nature.

[17] Sánchez Goñi MF, Landais A, Fletcher WJ, Naughton F, Desprat S (2008). Contrasting impacts of Dansgaard-Oeschger events over a western European latitudinal transect modulated by orbital parameters.. Quat Sci Rev.

[18] Salgueiro E, Voelker AHL, de Abreu L, Abrantes F, Meggers H (2010). Temperature and productivity changes off the western Iberian margin during the last 150 kyr.. Quat Sci Rev.

[19] Laquay G (1981). Recherches sur les faunes du Würm I en Périgord..

[20] Guadelli JL (1987). Contribution à l'étude des zoocoenoses préhistoriques en Aquitaine (Würm Ancien et Interstade Würmien)..

[21] Mourer-Chauviré C (1975). Les oiseaux du Pléistocène moyen et supérieur de France..

[22] Urich K (1994). Comparative animal biochemistry.

[23] Jeannet M (2010). http://pm.revues.org/index492.html.

[24] Faivre JP (2006). L'industrie moustérienne du niveau Ks (locus 1) des Fieux (Miers, Lot): Mobilité humaine et diversité des compétences techniques.. Bull Soc Preh Fr.

[25] Gerbe M, Thiébault C, Mourre V, Bruxelles L, Coudenneau A (2012). Influence des facteurs environnementaux, économiques et culturels sur les modalités d'exploitation des ressources organiques et minérales par les Néandertaliens des Fieux (Miers, Lot)..

[26] Discamps E, Jaubert J, Bachellerie F (2011). Human choices and environmental constraints: Deciphering the variability of large game procurement from Mousterian to Aurignacian times (MIS 5-3) in southwestern France.. Quat Sci Rev.

[27] Dibble HL, Berna F, Goldberg P, McPherron SP, Mentzer S (2009). A preliminary report on Pech de l'Azé IV, layer 8 (Middle Paleolithic, France).. PaleoAnthropology.

[28] Gaudzinski S, Niven L, Hublin JJ, Richards MP (2009). Hominin subsistence patterns during the Middle and Late Pleistocene in northwestern Europe.. The evolution of hominin diets: Integrating approches to the study of Palaeolithic subsistence.

[29] Roger T (2004). L'avifaune du Pléistocène moyen et supérieur du bord de la Méditerranée européenne: Orgnac 3, Lazaret (France), Caverna delle Fate, Arma delle Manie (Italie), Kalamakia (Grèce), Karain E (Turquie)..

[30] Soressi M, Rendu W, Texier JP, Claud E, Daulny L (2008). Pech-de-l'Azé I (Dordogne, France): Nouveau regard sur un gisement moustérien de tradition acheuléenne connu depuis le XIXe siècle..

[31] Juanes F (1986). Population density and body size in birds.. Am Nat.

[32] Kuhn SL, Belfer-Cohen A, Barzilai O, Stiner MC, Kerry KW (2004). The last glacial maximum at Meged Rockshelter, Upper Galilee, Israel.. Journal Israel Prehist Soc.

